# Infectious Agents As Markers of Human Migration toward the Amazon Region of Brazil

**DOI:** 10.3389/fmicb.2017.01663

**Published:** 2017-08-31

**Authors:** Ricardo Ishak, Luiz F. A. Machado, Izaura Cayres-Vallinoto, Marluísa de O. Guimarães Ishak, Antonio C. R. Vallinoto

**Affiliations:** Virus Laboratory, Institute of Biological Sciences, Federal University of Para Belem, Brazil

**Keywords:** biomarkers, infectious agents, epidemiology, human migration, Amazon

## Abstract

Infectious agents are common companions of humans and since ancient times they follow human migration on their search for a better place to live. The study of paleomicrobiology was significantly improved in its accuracy of measurement with the constant development of better methods to detect and analyze nucleic acids. Human tissues are constantly used to trace ancient infections and the association of anthropological evidences are important to confirm the microbiological information. Infectious agents which establish human persistent infections are particularly useful to trace human migrations. In the present article, the evidence of infection by viral agents such as human T-lymphotropic virus 1, human T-lymphotropic virus 2, human herpes virus-8, JC virus, and a bacterium, *Chlamydia trachomatis*, was described using different methodologies for their detection. Their presence was further used as biomarkers associated with anthropological and other relevant information to trace human migration into the Amazon region of Brazil. The approach also evidenced their microbiological origin, emergence, evolution, and spreading. The information obtained confirms much of the archeological information available tracing ancient and more recent human migration into this particular geographical region. In this article, the paleomicrobiological information on the subject was summarized and reviewed.

## Introduction

Viral agents are commonly associated to infections which are able to trace human ancient and recent migrations all over the world. In the early history of civilization small human groups started to move around the world to find safer environment and food. Their traveling into previously unknown areas brought also the interaction with new infectious agents. Recent human spread as a consequence of wars, famine, disasters, and many other modern dangers also carried old and new agents with greater or less chance to spread and cause severe prejudice.

It was not until recently that we could seriously evaluate the effect of emerging infectious agents in the course of the human migration around the world. The improvement of nucleic acid analysis facilitated the detection of ancient molecules of infectious agents which was an enormous step toward the delineation of historical patterns of infections and ancient migrations of human populations. The study of paleomicrobiology was significantly advanced with the detection of the DNA of *Mycobacterium tuberculosis* in an ancient skeleton which progressed with the identification of infectious agents and their geographical spreading, as they were related to their respective paleo specimens (Spigelman and Lemma, 1993; Raoult and Drancourt, 2008; Djelouadji et al., 2011).

Dental pulp specimens allowed the recovery of human immunodeficiency virus (HIV-1) integrated DNA from fibroblasts of an AIDS patient (Glick et al., 1991), as well as the DNA of *Yersinia pestis* from skeletons dated from the 14th, 16th, and 18th centuries (Drancourt et al., 1998; Raoult et al., 2000). Human papillomaviruses and human T-lymphotropic virus 1 (HTLV-1) genetic materials were detected in tissues dated from the 4th (Li et al., 1999) and 16th (Fornaciari et al., 2003) centuries. Most of the time such approaches are better suited to viruses which generally establish persistent infections, but influenza virus (H1N1), a short replication cycle RNA virus was described in paraffin embedded biopsies from the 1918 epidemic (Reid et al., 1999). More recently, hepatitis C virus (HCV), an RNA virus which is able to establish persistence in humans without nucleic acid integration, was recovered from the dental pulp and could be used as a biomarker of past and ancient infections (Siravenha et al., 2016).

Persistence of viruses allows the application of molecular biology methods, but also of other methods; antibody detection using serological studies are indicative of the presence of the persistent virus infection. This approach coupled to molecular biology methods has allowed us to define the presence of past and old infections among specific population groups in the Amazon region of Brazil within epidemiologically closed groups of “quilombolas” (slave refugees) or native Indians and immigrants with little or no degree of miscegenation at all.

Epidemiological studies, mostly descriptive ones, were important biological tools when associated to molecular biology approaches, particularly when dealing with the recent history of specific infectious agents. Besides describing the presence of the infectious agent, it also showed the valuable importance of surveillance when detecting the recent entry of new infectious agents among human population communities. In the recent history of the study of infectious agents in the Amazon region of Brazil, there are some examples of major relevance, including the involvement of native Indians with devastating agents such as hepatitis B virus (HBV) and HIV-1 (Santos et al., 1995; Machado et al., 2006; Nunes et al., 2007).

An extensive seroepidemiological study was performed with native Indians looking for the presence of infection markers of HBV. There was one community, namely the Parakana, which was found to be free of the agent. The main characteristic was that the samples were collected within 2 weeks after the initial contact with the community (Santos et al., 1995). Few years only were necessary to show the introduction and rapid spreading of the virus in the Parakana (Nunes et al., 2007). A situation which was shown to be even more critical and distressing was shown by our laboratory concerning the recent entry of HIV-1 within an Indian community, the Tiryio, who reside in the North region of the Amazon. Retrospective seroepidemiology with historical samples from different Indian communities showed that the virus was not present until the early 1990s (Ishak et al., 1989; Zaninovic et al., 1994; Machado et al., 2006), but it was readily introduced most probably as a sexually transmitted infection (Machado et al., 2006) as usually seen with other viruses and bacteria regarding native Indian communities (Ishak and Ishak, 2001; Machado et al., 2006). These are common examples of the entry and spreading of infectious agents affecting previously epidemiologically closed and isolated Indian groups, threatened by sexually transmitted viruses and bacteria that can act as real dangers to their fertility and life span. The present article is a comprehensive review of the existent information in the field of paleomicrobiology relating the evidences of the most probable routes of how viral and bacterial infectious agents entered in the Amazon region of Brazil in ancient and more recent times together with human beings moving toward this environment.

## Methodological Approaches

The initial general approach for studying viruses and their relation to human migrations investigated the presence of antibodies. As we were dealing with agents which established persistent infections the positive reactions were also a confirmation of the presence of the virus in the human host. The following step was to generate information of the genotypes, or serotypes, circulating in the Amazon region of Brazil and to associate them with previously described geographical distribution of the virus and their variants. The approach for the study of *Chlamydia trachomatis* was similar, as the bacterium is thought to persist in the infected persons. The evaluation of the antibody response to the serotypes indicated their spreading. The specific methodological approach for each infectious agents is reported briefly below. Specific details are described in the original publications.

### HTLV

**FIGURE 1:**
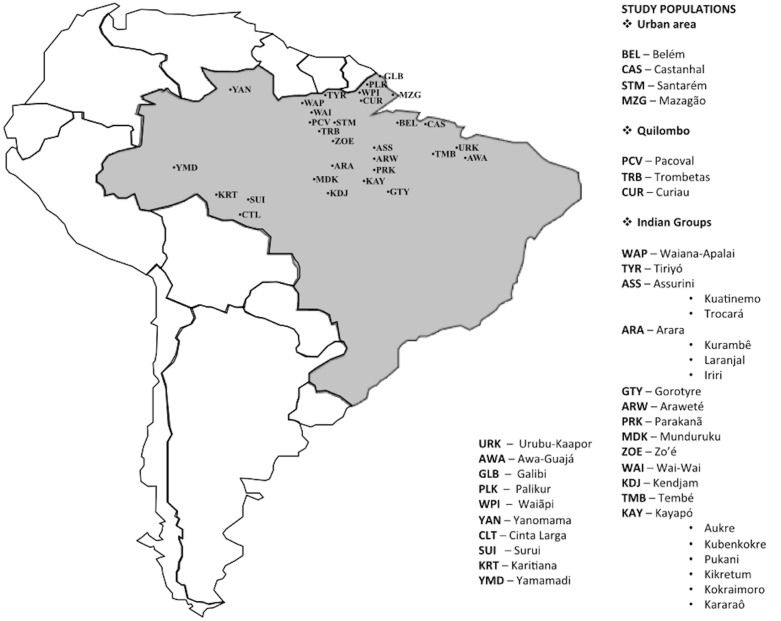
Geographical location of the human populations investigated for HTLV, JCV, HHV-8, and *Chlamydia* infections (prepared by the authors and edited using Power Point for Mac).

Antibodies to HTLV-1 and HTLV-2 were detected using an enzyme immune assay (and confirmed using a commercial Western blot) in serum samples of 26 native Indian tribes largely distributed in the Amazon region of Brazil (**Figure 1**), belonging to communities from the States of Maranhao (Urubu-Kaapor), Amapa (Galibi, Palikur, Waiapi), Roraima (Yanomami), Amazonas (Yamamadi), Rondonia (Cinta Larga, Surui, Karitiana), and Para (Wayana-Apalai, Tiryio, Assurini do Kuatinemo, Assurini do Trocara, Arara do Laranjal, Arara do Kurambe, Arara do Iriri, Gorotire, Arawete, Parakana, Munduruku, and six villages of the Kayapo, namely the Kararao, Aukre, Kubenkokre, Pukany, Kikretum, and Kokraimoro). Nucleic acid extraction was performed from positive samples in order to sequence regions of the gene *env* and LTR region to construct phylogenetic trees which allowed to describe the molecular subtype HTLV-2c. Detailed methodological steps were previously described (Ishak et al., 1995; Vallinoto et al., 2002).

### JCV

The initial approach to investigate JC virus (JCV) in the Brazilian Amazon involved 10 native Indian tribes from the States of Para (Wai-Wai, Munduruku, Kendjam, Arara Laranjal, Parakana, Aukre, Tembe, and Assurini), Maranhao (Urubu-Kaapor), and Rondonia (Surui), one Brazilian afro-descendant “quilombo” from the Rio Trombetas region in the West of the State of Para and from its capital, the city of Belem (**Figure 1**). JC virus was detected in urine samples using the amplification of *vp1* gene and IG region according to a previously described protocol (Cayres-Vallinoto et al., 2012). The amplified products of the IG region were used to construct the phylogenetic trees and subsequent relationships among the strains detected. Detailed methodological steps were previously described (Cayres-Vallinoto et al., 2012).

### HHV-8

Antibodies to human herpes virus-8 (HHV-8) were investigated among four native Indian groups (Kararao, Arara Laranjal, Tiriyo, and Zo’e) from the Amazon region (**Figure 1**). An enzyme immune assay with four structural and non-structural antigens (Katano et al., 2000) was used. Nucleic acid extraction was performed in the positive samples in order to sequence region *ORF26* and region 1 of the *k1* gene to confirm serological results and to prepare phylogenetic trees which allowed to describe the molecular subtypes of HHV-8. Detailed methodological steps were previously described (Chang et al., 1994; Cook et al., 1999; Ishak et al., 2007).

### Chlamydia trachomatis

Antibodies to *Chlamydia* were detected in the blood samples of: (i) patients from a university public hospital (respiratory and cardiac disease) in the city of Belem, Para; (ii) subjects from two major urban communities (Santarem and Castanhal) of the State of Para; (iii) individuals from two isolated Afro-descendant communities located in Northern Brazil (Pacoval, State of Para, and Curiau, State of Amapa) who originated from runaway individuals during the slavery period; (iv) individuals from Mazagao, State of Amapa, a village originating from immigrants of North Africa; and (v) three native Indian groups from the State of Maranhao (Awa-Guaja), and the State of Para (Wayana-Apalai and Kokraimoro) (**Figure 1**). A commercial indirect immunofluorescence assay using serotype L2 of *C. trachomatis* as the antigen was used for the screening of antibodies to the genus *Chlamydia*. Positive samples were titrated and those with titers equal or greater than 512 were investigated to detect serological evidence of bacterium persistence (Conway et al., 1984; Lobos et al., 1996). Serum reactivity to specific serotypes of *C. trachomatis* was discriminated using a microimmunofluorescence assay with serially diluted samples to detect the most recent infection, as well as double or multiple infections. Detailed methodological steps were previously described (Wang and Grayston, 1974; Grayston and Wang, 1975; Treharne et al., 1983; Conway et al., 1984; Ishak et al., 1988, 1993, 2015; Campbell et al., 1990; Lobos et al., 1996; Ishak and Ishak, 2001).

## The Evidences of Human Migration Accompanied by Infectious Agents in the Amazon Region of Brazil

### HTLV

**FIGURE 2:**
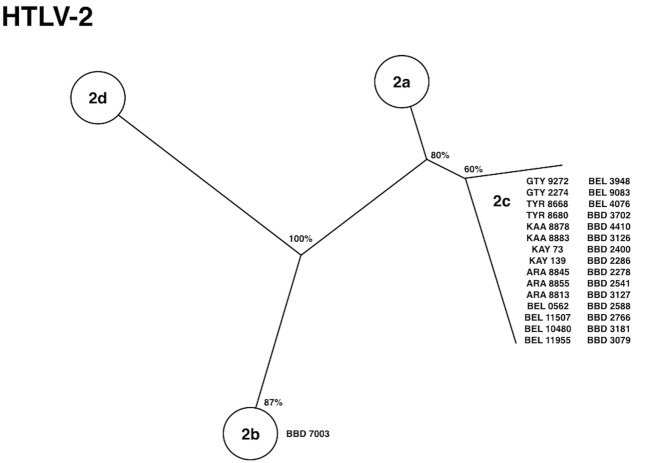
Phylogenetic tree showing HTLV-2 subtypes isolated from native Indian communities from the Amazon region of Brazil [adapted from Ishak et al. (1995) and Vallinoto et al. (2002)].

The initial investigation of HTLV among Indian populations in the Amazon region of Brazil was based on retrospective seroepidemiological studies aiming to find previous infection markers of the virus among human populations residing in this particular area. Previously, HTLV-2 infection was shown to be present among few individuals of the Kayapo and Kraho villages (Maloney et al., 1992). Subsequently, our group described the serological and molecular epidemiology of HTLV infection among 18 native Indian tribes located in the Brazilian Amazon region, and the existence of a unique molecular subtype named HTLV-2c (**Figure 2**). This subtype is endemically distributed in a large geographical setting (more than 5 million km^2^) and maintained under continuous transmission (vertical and horizontally) among epidemiologically isolated human groups due to their physical, cultural, and linguistic barriers (Ishak et al., 1995; Eiraku et al., 1996). The virus was later confirmed in other Amerindian communities and in different groups (blood donors and HIV-1 infected patients) residing in the city of Belem, the largest urban area in the Amazon region (Vallinoto et al., 2002).

**FIGURE 3:**
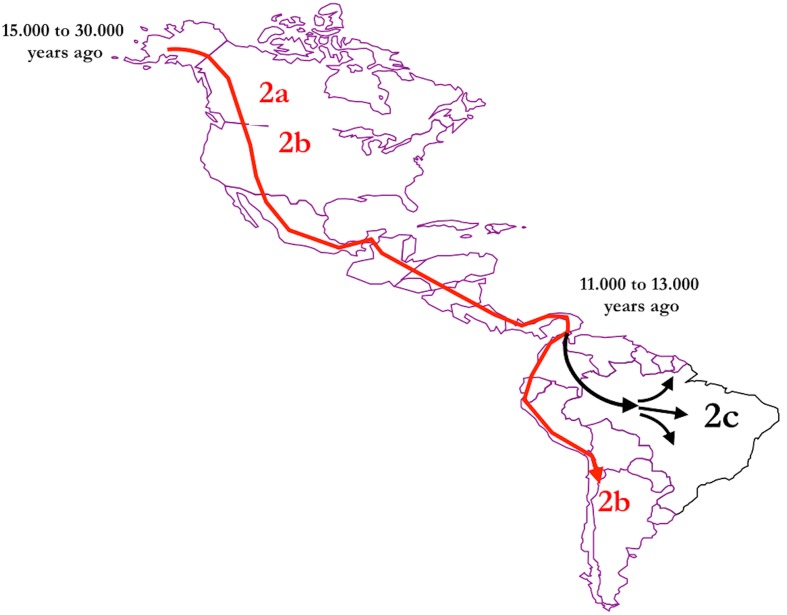
Possible route of HTLV-2 dissemination and origin of HTLV-2c according to the ancient human migration in the American continent (prepared by the authors and edited using Power Point for Mac).

The geographical distribution of HTLV-2 around the world shows that in the Americas, the virus is present from North to South America in the molecular subtypes HTLV-2a (predominantly in urban areas) and HTLV-2b, predominantly among native Indians groups from North, Central, and South America (Heneine et al., 1991; Biglione et al., 1993; Ferrer et al., 1993; Levine et al., 1993; Switzer et al., 1995). The distribution of molecular subtype HTLV-2c follows another pattern and is largely spread among Amerindians from the Amazon region of Brazil. Human T-lymphotropic virus 2 has apparently emerged in the African continent and was brought to the Americas, crossing the Bering Strait probably 11,000–13,000 years ago (Switzer et al., 1996; Vallinoto et al., 2002). Archeological, anthropological, and genetic studies have shown that a wave of migration occurred apart from the Andean region, to the Amazon region in South America, which may have introduced HTLV-2c exclusively into the region and evolved independently from the other subtypes (**Figure 3**).

Once the virus reached its way into the community it was actively transmitted horizontally (via the sexual route) and maintained within epidemiologically closed communities using the vertical transmission from mother to child, a characteristic of retroviral persistent infections with integration of the viral nucleic acid into the host genome (Ishak et al., 1995, 2001; Vallinoto et al., 2002). The widespread distribution among indigenous populations with different linguistic and cultural backgrounds is the main support for the introduction and spreading of the virus in ancient populations. Genetic processes of fission and fusion of large and small communities, respectively, were probably responsible for the current widespread distribution of the virus. The presence of the virus in urban communities is a direct result of the extensive miscegenation of the Brazilian population which occurred during the colonization period. It is possible that the introduction of HTLV-2c is a consequence of an ancient process occurring over many generations. The lack of serological evidence of previous contact with HTLV in some groups suggests the occurrence of the founder effect and the introduction in urban areas as a recent event through sexual transmission (Ishak et al., 1995; Vallinoto et al., 2002).

More recently, the virus was detected in several major cities of Brazil, including Salvador (Barreto et al., 2014), Belo Horizonte (Catalan-Soares et al., 2005), Rio de Janeiro (Silva et al., 2002), São Paulo (Eiraku et al., 1996), and Porto Alegre (Renner et al., 2006). The wide distribution of the virus from North to South of the country is a result of a more recent spreading of the virus from the Indian communities to urban areas and, subsequently, into highly populated cities. The virus was also detected among Guarani Indians in the far South of Brazil, in the State of Parana (Menna-Barreto et al., 2005).

**FIGURE 4:**
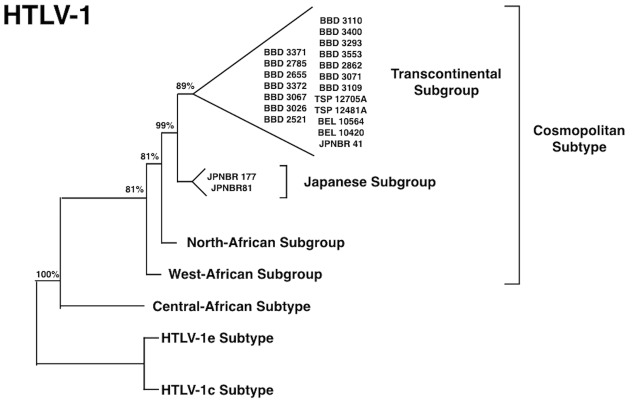
Phylogenetic tree showing HTLV-1 subtypes isolated from Japanese immigrants in the Amazon region of Brazil [adapted from Vallinoto et al. (2004)].

**FIGURE 5:**
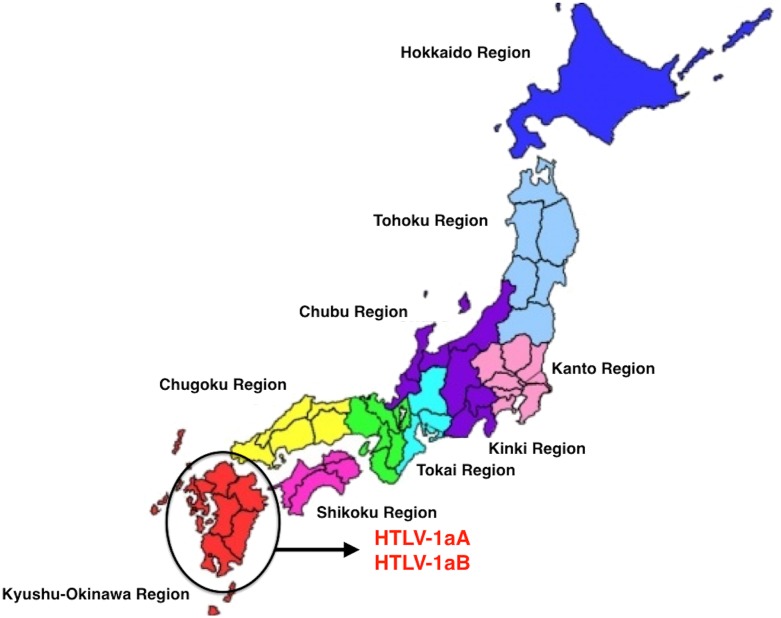
Geographical origin of the Japanese immigrants who brought HTLV-1 subtypes, living in Para State, Northern Brazil (prepared by the authors and edited using Power Point for Mac).

Brazil received large waves of migration around 100 years ago, in the beginning of the 20th century, from European nations (particularly from Portugal, Spain, and Italy) and at a smaller scale from Japan (Emmi, 2013). Our group was able to trace the migration and the introduction of a new virus, HTLV-1, into the Amazon region, following this more recent pathway. The virus is highly prevalent in the South of Japan (Yamashita et al., 1999) and a great amount of Japanese immigrants were brought to the North of Brazil (State of Para) and were kept as a small epidemiologically closed community, with their original social, cultural, and behavior for decades. Human T-lymphotropic virus 1 was probably introduced in the Amazon region, on two different occasions during human colonization. In the first move, the African descendants brought the virus to Brazil during the slavery period and, more recently during a second wave of migration from Japan. The presence of HTLV-1, subtypes Transcontinental and Japanese, among immigrants living in a small rural city named Tome-Acu (Para State, Northern Brazil) was clearly characterized (Vallinoto et al., 2004; **Figure 4**). Tome-Acu was a village settled by the Japanese immigrants for specific agricultural purposes (Emmi, 2013). The infected persons were three women who were more than 50 years old, and migrated from the Kyushu region, Southeast of Japan (**Figure 5**). The virus kept a similar genetic sequence as imported from Japan.

### JCV

**FIGURE 6:**
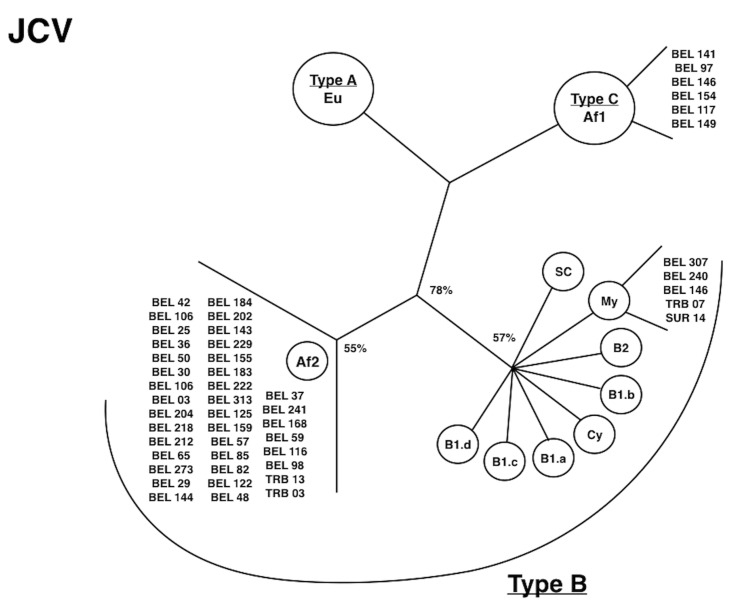
Phylogenetic tree showing JCV subtypes isolated from Amazon populations. [adapted from Cayres-Vallinoto et al. (2012)].

The finding of polyomavirus infections in the Amazon region helped to show the close association of migration routes of viruses and humans during their migration pathways. JC virus was described in the following communities: types A (subtype EU), B (subtypes Af-2, African and MY, Asiatic), and C (subtype Af-1) among subjects living in Belem; type B, subtypes Af-2 and MY, among Afro-Brazilians; and type B, subtype MY, within the Surui Indians (**Figure 6**; Cayres-Vallinoto et al., 2012).

Detection of the virus within an epidemiologically semi-closed population of native Indians reinforced the possibility of using JCV as a good marker for human migration in the Americas, including Canada and the United States (Stoner et al., 2000; Sugimoto et al., 2002). Prevalence studies of the virus in those countries are similar to those found in other countries of the Pacific and the Asian continent (Kunitake et al., 1995; Agostini et al., 1997; Stoner et al., 2000; Whiley et al., 2001, 2004; Sugimoto et al., 2002). In Belem, three ancestral types according to its geographical association were described: type A (found in Europe), type B (found in Africa and Asia), and type C (found in the African continent). This is in agreement with the historical miscegenetic process of the urban population of Brazil and the available information of the genetic composition of human populations living in Belem (Santos and Guerreiro, 1995; Rodrigues, 1999). The most frequent subtype (Af2) was present in samples from “mesticos,” a phenotype characteristically related to a black ethnicity. This is an important observation when considering that most of the Af2 genotype evolved within the African continent circa 50,000 years ago (Takasaka et al., 2006). Subtype MY from Belem was also detected among “mesticos,” a common finding among native Amerindians (Zheng et al., 2003). MY and Af2 were detected in the Trombetas “quilombo.”

The situation observed among urban areas, native Indians, and native Americans strongly suggests that JCV, type B, and subtype MY established persistent infections in humans and entered in a similar fashion as HTLV-2, into the Brazilian Amazonian population circa 10,000 years ago. However, a different route of JCV entry was probably more recent (200–300 years ago) during the slavery trade into Brazil. A second spread occurred in the “quilombos,” which occurred as early as in 1788 in the State of Para (Salles, 1998). This is shown by the extensive distribution of type B, subtype Af2 among the trihybrid groups of humans in the Amazon region of Brazil. Usually, a higher prevalence of JCV is observed in the Americas than on the African continent (Chima et al., 1998; Stoner et al., 2000), a possible consequence of the intense miscegenation of Afro-descendants in Brazil and in the United States. The HIV-1 epidemic was also followed by a marked spreading of JCV among urban communities (Nali et al., 2012; Cayres-Vallinoto et al., 2016).

### HHV-8

Human herpes virus-8 infections in the Amazon region showed a widespread agent both in urban and non-urban communities, including native Indians in Brazil, French Guiana, and Equador (Freitas et al., 2002; Whitby et al., 2004; Cunha et al., 2005; Kazanji et al., 2005; Ishak et al., 2007). The high positive percentage levels of antibodies found in some investigated communities indicated that vertical transmission was occurring and playing an important role for the virus maintenance and spread, as a herpesvirus persistent infection. There was a sharp and steady constant rise in the age prevalence of antibodies seen in the Indian villages of Kararao, Arara Laranjal, Tiriyo, and Zo’e, followed by a decrease in the prevalence after the first 10 years of life.

**FIGURE 7:**
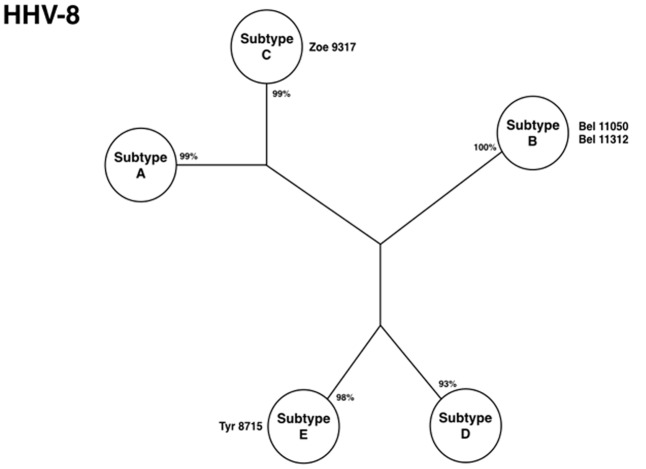
Phylogenetic tree showing HHV-8 subtypes isolated from Amazon populations [adapted from Ishak et al. (2007)].

Sequencing the VR1 region (gene *k1*) identified diversity of HHV-8 subtypes among urban and native Indians. Subtypes E and C were described in the Tiriyo and the Zo’e, respectively (**Figure 7**). Human herpes virus-8, subtype E, was also described among two other Brazilian Indian communities (Arawete and Assurini), in French Guiana (Wayampi), and in Equador (Biggar et al., 2000; Whitby et al., 2004; Kazanji et al., 2005). It emerged as a unique molecular subtype among Amerindians of the Amazon region with different linguistic groups. Subtype C is commonly associated to patients with classical Kaposi sarcoma (KS), iatrogenic, and KS present in AIDS patients in Middle East and Asia (Zong et al., 1999), and its finding in an epidemiologically closed community in the Amazon region of Brazil was quite exquisite, considering that those persons have never left their villages nor had any relation with outsiders.

The higher prevalence of HHV-8 among rural Amerindians than in urban areas, the presence of a diversity of molecular subtypes (three out of five, considering the urban areas), and the presence of a unique strain found solely within the Amazon region are strong evidence that the virus represents an ancient endemic infection of native Indians. Human herpes virus-8 is another example of how viruses which establish persistence can also help to understand the mobility of humans around the world in their search for new areas for a better living.

### Chlamydia trachomatis

The political organization of Brazil dates from the 1500s, starting as a colony of Portugal, but with several invasions throughout the centuries from other European countries, including Spain, Netherlands, and France. Several other waves of immigrants were also reported and some remain unclear. *C. trachomatis*, a widespread infection all over the world, is a bacterium used by our group to describe a more recent massive migration that occurred in the 18th century.

The presence of serotype A of *C. trachomatis* has been detected by our group, indirectly on several occasions among urban and non-urban communities including an extensive search among native Indians in the Amazon region of Brazil (Ishak and Ishak, 2001; Ishak et al., 2015). More recently, reactivity to serotype A was also described among patients in six different groups tested (in an urban university hospital, in some major cities of the State of Para, among native Indians, one Afro-descendant community, and particularly, among one village in the State of Amapa, named Mazagao). The village of Mazagao was established during the 18th century as a consequence of a massive immigration movement (more than 1,000 Portuguese Catholics) from North Africa. These people emigrated from North Africa to escape Muslim persecution, following a religious war that occurred in their hometown, a place also called Mazagao.

Trachoma an eye infection and a severe inflammatory disease caused by *C. trachomatis* is associated with infections by serotype A, usually described to occur within a limited area in the Middle East and Northern Africa, serotypes B and C commonly described as cosmopolitan strains and serotype B_1_ infecting North American Indians. The most probable explanation to establish the route of entry of serotype A into the Amazon region of Brazil was through the refugees from Mazagao. Since then, the bacterium was spread urban and non-urban populations elsewhere (through bad hygienic habits, poor conditions of living, and vectors among others). Curiously, serorreactivity to serotypes E and J, strains usually sexually transmitted, were seen exclusively within the Mazagao village.

## Conclusion

The different methods by which infectious agents are detected in ancient specimens were used in a combined approach with archeology to detect past and ancient infections in order to improve the understanding of the origin, emergence, evolution, and spreading of viruses, bacteria, and other infectious agents. Similar methods were also used to describe recent emergence and introduction of infectious agents.

In our previous studies, HTLV-2, JCV, HHV-8, and *C. trachomatis* were described among Amerindian tribes, Afro-descendant communities, and in the trihybrid population living in the urban and non-urban areas of the Amazon region of Brazil. The presence of different and largely variable strains of those agents circulating in the region and infecting selected human host groups brought light to the association of the ethnic origin of the populations investigated. The molecular linkage of different strains to specific areas in the European, Asiatic, and African continents is excellent tools to highlight the role of infectious agents as markers for studying the early migration of human populations, reflecting their early and late history.

The present world situation of political instability in several countries is, again, causing a massive movement of humans from Haiti to Brazil and from the Middle East to Europe (and to a lesser extent to other continents). It is quite certain that we will soon observe the introduction and upsurge of viruses and other infectious agents into different geographical areas. The use of infectious agents capable of establishing persistence is of major importance as models to study their clinical interaction with humans and microbiological active surveillance will be necessary to follow up the present ongoing migratory wave.

## Author Contributions

All authors listed have made a substantial, direct and intellectual contribution to the work, and approved it for publication.

## Conflict of Interest Statement

The authors declare that the research was conducted in the absence of any commercial or financial relationships that could be construed as a potential conflict of interest.
